# CXCR3 Expression Pattern on CD4+ T Cells and IP-10 Levels with Regard to the HIV-1 Reservoir in the Gut-Associated Lymphatic Tissue

**DOI:** 10.3390/pathogens11040483

**Published:** 2022-04-18

**Authors:** Max Augustin, Carola Horn, Meryem Seda Ercanoglu, Ute Sandaradura de Silva, Vincent Bondet, Isabelle Suarez, Seung-Hun Chon, Dirk Nierhoff, Elena Knops, Eva Heger, Carlo Vivaldi, Hartmut Schäfer, Mark Oette, Gerd Fätkenheuer, Florian Klein, Darragh Duffy, Michaela Müller-Trutwin, Clara Lehmann

**Affiliations:** 1 Division of Infectious Diseases, Department I of Internal Medicine, Medical Faculty and University Hospital Cologne, University of Cologne, 50937 Cologne, Germany; max.augustin@uk-koeln.de (M.A.); carola.horn@uk-koeln.de (C.H.); ute.sandaradura-de-silva@uk-koeln.de (U.S.d.S.); isabelle.suarez@uk-koeln.de (I.S.); gerd.faetkenheuer@uk-koeln.de (G.F.); 2 Center for Molecular Medicine Cologne (CMMC), Medical Faculty and University Hospital Cologne, University of Cologne, 50937 Cologne, Germany; meryem.ercanoglu@uk-koeln.de (M.S.E.); florian.klein@uk-koeln.de (F.K.); 3 German Center for Infection Research (DZIF), Partner Site Bonn-Cologne, 50931 Cologne, Germany; 4 Institute of Virology, Medical Faculty and University Hospital Cologne, University of Cologne, 50937 Cologne, Germany; elena.knops@uk-koeln.de (E.K.); eva.heger@uk-koeln.de (E.H.); 5 Translational Immunology Unit, Institut Pasteur, Université de Paris, CEDEX 15, 75015 Paris, France; vincent.bondet@pasteur.fr (V.B.); darragh.duffy@pasteur.fr (D.D.); 6 Department of General, Visceral Surgery and Cancer Surgery, Medical Faculty and University Hospital Cologne, University of Cologne, 50937 Cologne, Germany; seung-hun.chon@uk-koeln.de; 7 Clinic for Gastroenterology and Hepatology, Medical Faculty and University Hospital Cologne, University of Cologne, 50937 Cologne, Germany; dirk.nierhoff@uk-koeln.de; 8 Clinic for Coloproctology, PanKlinik, 50667 Cologne, Germany; c.vivaldi@enddarmpraxis-koeln.de (C.V.); h.schaefer@enddarmpraxis-koeln.de (H.S.); 9 Department of General Medicine, Gastroenterology and Infectious Diseases, Augustinerinnen Hospital, 50678 Cologne, Germany; moette@severinskloesterchen.de; 10 Unité HIV, Inflammation & Persistence, Institut Pasteur, Université Paris Cité, CEDEX 15, 75015 Paris, France; mmuller@pasteur.fr

**Keywords:** HIV reservoir, GALT, cART, CXCR3, IP-10, immune activation, memory CD4+ T cell subsets

## Abstract

(1) Background: The gut-associated lymphatic tissue (GALT) represents the largest lymphoid organ, and is considered to be the largest HIV reservoir. The exact size of the GALT reservoir remains unclear. Several markers, such as the chemokine receptor CXCR3 and its pro-inflammatory ligand IP-10, have been proposed to define the size of HIV reservoirs in the peripheral blood (PB). However, little is known about the role of CXCR3 and IP-10 within the GALT. (2) Methods: We compared the CXCR3 expression, IP-10 levels, and cell-associated HIV DNA of distinct memory CD4+ T cell subsets from the terminal ileum (TI), PB and rectum (RE) of 18 HIV+ patients with antiretroviral therapy (ART), 6 HIV+ treatment-naive patients and 16 healthy controls. (3) Results: While the relative distributions of CD4+ T cell subsets were similar in PB, TI and RE, HIV DNA and CXCR3 expression were markedly increased and IP-10 levels were decreased in TI when compared to PB. No significant correlation was found between the CXCR3 expression and memory CD4+ T cell subsets, IP-10 levels and the HIV DNA amounts measured in PB, TI or RE. (4) Conclusions: During a chronic HIV-1 infection, neither CXCR3 nor IP-10 are indicative of the size of the viral reservoir in the GALT (TI and RE).

## 1. Introduction

Despite combined antiretroviral therapy (cART), human immunodeficiency virus (HIV-1) reservoirs of the gut-associated lymphatic tissue (GALT) are very difficult to cure [1]. A particularly large, localized collection of lymphocytes can be found in the terminal ileum (TI), organized in so-called Peyer’s patches [2,3]. In this context, it is essential to understand how large the reservoir is, especially in the GALT. However, sampling biopsies from TI is challenging, which limits our knowledge of the HIV cellular reservoir in TI. The current understanding of the human GALT results from research into human rectal tissues (RE) or animal models [4,5,6,7]. Nevertheless, this does not adequately reflect the real conditions in TI.

Several markers have been proposed to define the sizes of cellular HIV reservoirs. The first, and probably best known, is the surface marker CD32. After its first scientific description in the context of HIV reservoirs in 2017 [8], several studies discordantly discussed CD32 as a reliable marker [9,10,11]. However, CD32 re-emerged as a marker of HIV reservoirs in a highly purified cell population in 2020 [12]. To date, the role of CD32 in HIV latency remains controversial. The second is the chemokine receptor CXCR3 and its pro-inflammatory ligand IP-10 [13,14]. In chronically SIV-infected non-human primates (NHP), CXCR3 and IP-10 were found more frequently in the GALT than in the peripheral blood (PB) [14], and have been associated with the size of the viral reservoir. CXCR3 is preferentially expressed on Th1 cells, and when co-expressed with CCR6, it defines a subset of T_CD4_ that preferentially accumulates with HIV DNA in HIV-infected individuals on ART [15]. In HIV+ patients, blood IP-10 levels are increased and CXCR3 is up-regulated on memory CD4+ T cells [15,16,17]. In particular, the exposure of resting CD4+ T cells to chemokines, such as IP-10, allows efficient HIV-1 nuclear localization and integration of the HIV-1 provirus [18]. Thus, CXCR3/IP-10 may fuel peripheral and mucosal HIV-1 replication, and, therefore, chronic immune activation. In addition, replication-competent HI viruses were primarily found in CXCR3-expressing CD4+ T cells in the PB of HIV+ patients [19], and blood IP-10 levels correlate with the size of the viral reservoirs in the blood and semen [14,20]. CXCR3 and IP-10, which stimulate HIV target T cells to migrate to inflamed and peripheral tissues, are produced in high amounts in the small intestine [14] and stimulate HIV replication in vitro [15,21]. In addition, monoclonal blocking of CXCR3/IP-10 has been shown to reduce HIV-1 replication in vitro [21]. However, CXCR3’s role in latently infected memory CD4+ T cell subsets in the human terminal ileum and rectum remains unknown.

We, therefore, determined the levels and cellular distribution of HIV in the TI—the largest immune compartment in the GALT—in cART-treated (HIV^+^_cART_) versus treatment-naïve HIV-positive patients (HIV^+^_NAIVE_). We evaluated the local IP-10 levels and expression of the biomarkers CXCR3 on CD4+ T cell subsets, compared to the PB and GALT (TI + RE).

## 2. Results

### 2.1. Patient Characteristics

The median ages of the 18 cART-treated HIV-positive patients (HIV^+^_cART_) and treatment-naive HIV-positive patients (HIV^+^_NAIVE_) were 55 years (interquartile range(IQR) 47–62 years) and 49 years (IQR 42–50 years), respectively (Table 1). HIV^+^_cART_ had a CD4 cell count of 745 (IQR 464–973) per microliter (µL) and a plasma viral load below 20 ribonucleic acid (RNA) copies per milliliter (mL). In contrast, HIV^+^_NAIVE_ presented with a CD4+ cell count of 70/µL (IQR 20–270) per µL and a plasma viral load of 792,300 (IQR 17,280–3,700,000) copies/mL (Table 1).

### 2.2. Loss of CD4+ T Cells in GALT of All HIV+ Patients (cART-Treated and Treatment Naive)

**Figure 1:**
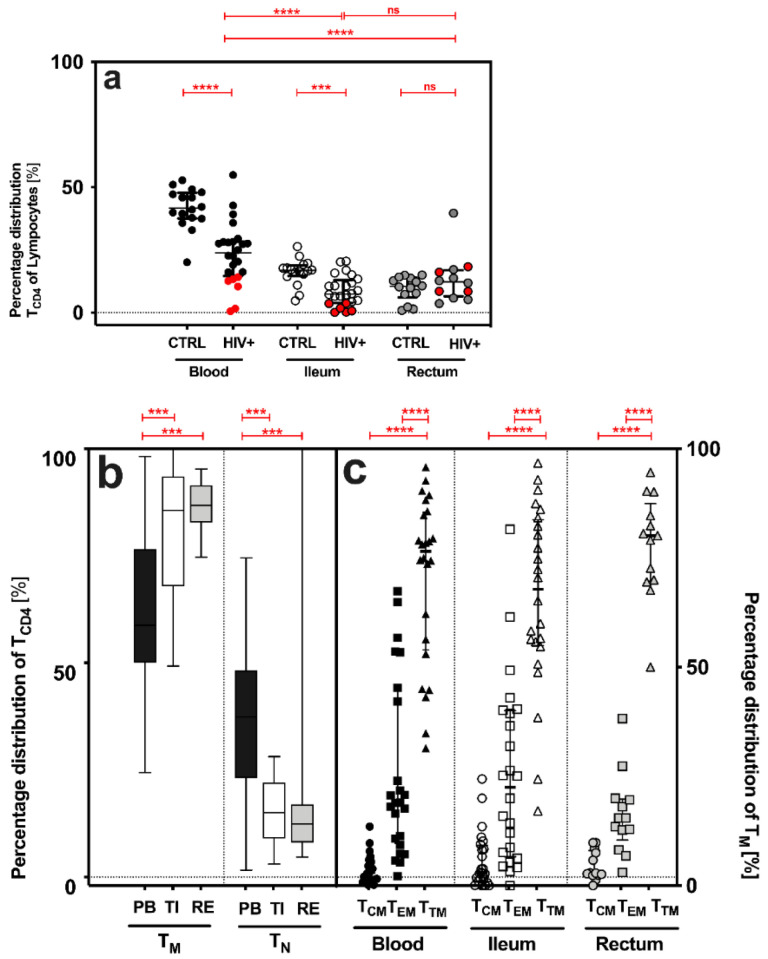
Quantitative features of CD4+ T cells (T_CD4_), memory (T_M_) and antigen-naive (T_N_), and distinct CD4+ T cell subsets (T_CM_, T_EM_ and T_TM_), in the peripheral blood (PB) and gut-associated lymphatic tissue (GALT = terminal ileum (TI) and rectum (RE)) of HIV+ and CTRL. (**a**) Percentage distribution [%] of CD4+ T cells (T_CD4_), cART-treated (HIV^+^_cART_, marked black) plus treatment-naive (HIV^+^_NAIVE_, marked red) HIV-positive patients versus control (CTRL). (**b**,**c**) Percentage distribution [%] of (**b**) memory (T_M_) and antigen-naïve (T_N_) CD4+ T cells and (**c**) distinct CD4+ T cell subsets (T_CM_, T_EM_ and T_TM_) in HIV+. Data are expressed as median (IQR) in graph (**a**,**c**), and minimum to maximum in graph b. Significant differences (*p*-values < 0.05) were tested with a two-tailed Mann-Whitney test or Wilcoxon matched-pairs test, as applicable. *p* < 0.05 shows statistical significance: *** *p* ≤ 0.001, **** *p* ≤ 0.0001, and ns, not significant. HIV+, all HIV-positive individuals (n = 24); CTRL, control; T_CM_, central memory T cells; T_EM_, effector memory T cells; T_TM_, transitional memory T cells; IQR, interquartile range; mL, milliliter.

CD4+ T cells (T_CD4_) were significantly decreased in (i) the PB and TI of HIV+ when compared to the CTRL (PB: *p* < 0.0001, TI: *p* = 0.0002, and RE: *p* = 0.3408) and (ii) the GALT of HIV+ when compared to PB (T_CD4_: PB vs. TI *p* < 0.0001, and PB vs. RE *p* = 0.0002) [% median (IQR), T_CD4_: HIV+: PB: 23.9 (14.6–28.3), TI: 7.4 (3.8–13.0), R: 10.5 (6.0–13.9); CTRL: PB: 41.7 (37.6–47.8), TI: 16.8 (4.6–18.8), R: 12.3 (6.5–16.9); Figure 1a].

### 2.3. Similar Distribution of Memory CD4+ T Cell Subsets in HIV+ and Healthy Individuals

**Figure 2:**
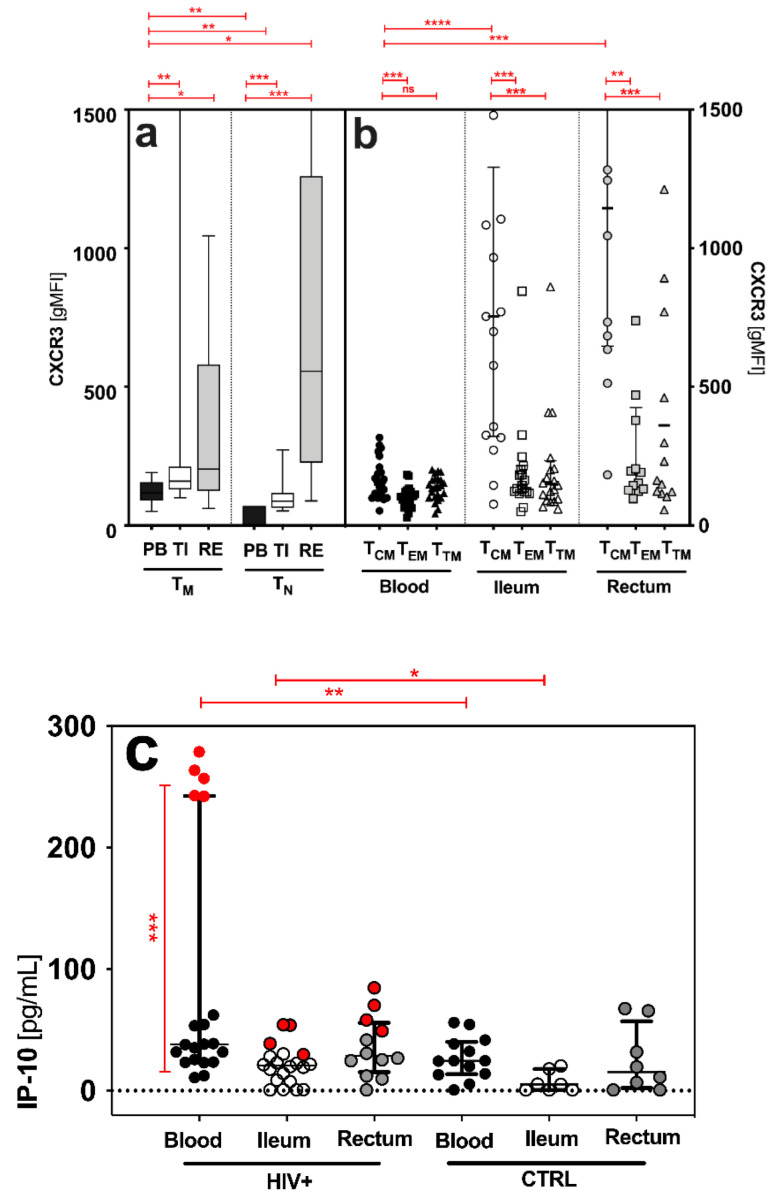

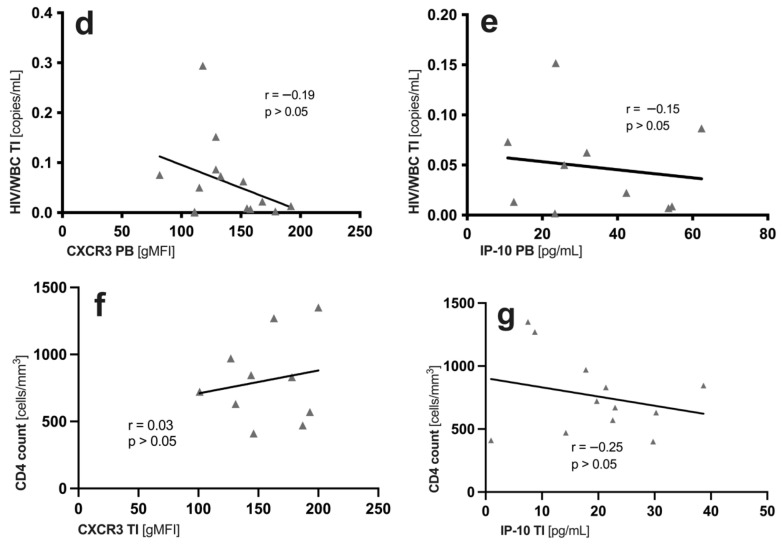
Qualitative features of memory (T_M_), antigen-naïve (T_N_) CD4+ T cells and distinct CD4+ T cell subsets (T_CM_, T_EM_ and T_TM_) in the peripheral blood (PB) and gut-associated lymphatic tissue (GALT = terminal ileum (TI) and rectum (RE)) of HIV+. CXCR3 expression [gMFI] on (**a**) antigen-naïve (T_N_) and memory (T_M_) CD4+ T cells and (**b**) distinct CD4+ T cell subsets (T_CM_, T_EM_ and T_TM_). (**c**) IP-10 levels in HIV+ and healthy controls in all sites. In HIV+ blood, HIV+_NAIVE_ are marked red. Correlations of (**d**,**e**) HIV DNA measured in the TI and (**f**,**g**) systemic CD4 cell count to ileal CXCR3 expression and ileal IP-10 levels, respectively. Data are expressed as median (IQR) in graphs a and c, and minimum to maximum in graph b. Significant differences (*p*-values < 0.05) were tested with a two-tailed Mann-Whitney test or Wilcoxon matched-pairs test, as applicable. *p* < 0.05 shows statistical significance: * *p* ≤ 0.05, ** *p* ≤ 0.01, *** *p* ≤ 0.001, **** *p* ≤ 0.0001, and ns, not significant. HIV^+^, HIV-positive individuals; cART, combined antiretroviral therapy; HIV^+^_NAIVE_, treatment-naïve HIV+; HIV^+^_cART_, cART-treated HIV^+^; T_CM_, central memory T cells; T_EM_, effector memory T cells; T_TM_, transitional memory T cells; gMFI, geometric mean fluorescence intensity; IQR, interquartile range; mL, milliliter; pg, picograms; PB, peripheral blood; TI, terminal ileum; WBC, white blood cells.

While the frequency of memory CD4+ T cells (T_M_) increased significantly from the PB to TI and RE, the frequencies of antigen-naive CD4+ T cells (T_N_) decreased (Figure 1b). Transitional memory T cells (T_TM_) were the most common memory cell subset in all three compartments (%, T_TM_-PB: 76.6 (53.9–85.5), TI: 67.9 (55.0–83.7), RE: 80.1 (69.8–87.5); Figure 2b), followed by effector memory (T_EM_) and central memory T cells (T_CM_): T_TM_ > T_EM_ > T_CM_ (Figure 1c).

### 2.4. Terminal Ileum (TI) Is the Preferential HIV DNA Reservoir

Similarly to our previous analyses [22], HIV^+^_cART_ harbored less HIV DNA compared to HIV^+^_NAIVE_ in all sites (PB: 21-fold, TI: 11-fold, and RE: 92-fold; not shown). However, the HIV DNA levels of solely HIV^+^_cART_ were significantly higher in (i) the GALT when compared to the PB (PB vs. TI + RE; *p* = 0.0327) and (ii) in the TI when compared to the RE (median (IQR) HIV/T_M_ × 10^6^, PB: 5866 (2098–11,855), TI: 13280 (5490–75,460), and RE: 5150 (0–32,280): TI > RE = PB; not shown). While T_EM_ carried the most HIV DNA in the PB (median (IQR) HIV/T_M_ × 10^6^, PB T_EM_: 1780 (1123–3890); T_CM_: 873 (128–1615); T_TM_: 642 (0–208)), T_TM_ did in the TI and RE (median (IQR) HIV/T_M_ × 10^6^ PB: T_EM_: 1780 (1123–3890), T_CM_: 873 (128–1615), and T_TM_: 642 (0–208); TI: T_TM_: 5490 (1650–34,700), T_EM_: 2960 (0–18,200), and T_CM_: 0 (0–5850); RE: T_CM_: 0 (0–17,700), T_EM_: 0 (0–2790), and T_TM_: 1510 (0–6080); not shown). The HIV-1 detection rates were, on average, 89%, 67%, and 67% in the PB, TI, and RE of HIV^+^_NAIVE_, and 80%, 70%, and 53%, respectively, of cART-treated patients.

### 2.5. Increased Expression of CXCR3 in the GALT of HIV+ Patients

In the PB, TI and RE, we found higher expression of CXCR3 on both antigen-naive T cells (T_N_: CD3+CD4+CD45RO−) and CD4+ T memory cells (T_M_: CD3+CD4+CD45RO+) in HIV+ patients compared to healthy controls (T_N_: CXCR3 2.7-fold increase and T_M_: CXCR3 1.7-fold increase; not shown). In addition, T_M_ showed significantly higher CXCR3 expression than T_N_ in the PB and TI (Figure 2a). A comparison of the sites showed that HIV+ patients had higher CXCR3 expression on T_M_ in the TI and RE than in the PB (median (IQR) of CXCR3 gMFI, T_M_: PB: 124 (89–157), TI: 160 (131–212), RE: 223 (128–685), TN: PB: 60 (45–78), TI: 88 (64–118), and RE: 557 (227–1261); Figure 2b). Central memory T cells (T_CM_) had the highest CXCR3 expression in all three compartments (Figure 2b). In addition, T_CM_ in the TI and RE showed significantly higher CXCR3 expression than the T_CM_ in the PB (Figure 2b).

### 2.6. No Correlation between IP-10/CXCR3 and CD4 Cell Count and Measured HIV DNA in the Blood and GALT of HIV^+^_cART_

The IP-10 levels were significantly higher in the HIV+ patients than in the healthy controls, and higher in the PB than in the GALT (Figure 2c). HIV^+^_NAIVE_ patients showed higher IP-10 levels in the PB than HIV^+^_cART_ (Figure 2c). We found no correlation (*p* > 0.05) between the CXCR3 expression on memory CD4+ T cell subsets or IP-10 levels and the levels of measured HIV DNA in the blood (CXCR3: r = −0.2, *p* > 0.05; IP-10: r = 0.09, *p* > 0.05); not shown) and the GALT (CXCR3: r = −0.19, *p* > 0.05; IP-10: r = −0.15, *p* > 0.05; Figure 2d,e). Importantly, clinical characteristics, such as CD4+ count (CXCR3: r = −0.2, *p* > 0.05; IP-10: r = 0.2, *p* > 0.05) and viral load (CXCR3: r = 0.00, *p* > 0.05; IP-10: r = 0.00, *p* > 0.05; not shown), had no impact on HIV DNA, IP-10 levels and CXCR3 expression in the blood (Table 1) and the GALT of HIV^+^_cART_ (CXCR3: r = 0.03, *p* > 0.05; IP-10: r = −0.25, *p* > 0.05; Figure 2f,g).

## 3. Discussion

A key mechanism in the pathogenesis of HIV infection is the gradual decline in CD4+ T cells (T_CD4_) [23], which occurs to a greater extent in the GALT [24,25,26] than in the PB [27,28,29]. It is also recognized that the gut is a large reservoir of HIV, but it is poorly quantified. Therefore, biomarkers such as CXCR3 and its ligand, the chemokine IP-10, may be helpful to gain a better understanding of the size of the reservoir. However, CXCR3’s and IP-10’s roles in latently infected memory CD4+ T cell subsets in the human terminal ileum and rectum remain unknown.

We, therefore, performed a comparative analysis of cell-associated HIV DNA, the expression of the biomarker CXCR3 on CD4+ T cell subsets, and IP-10 levels in the TI, PB, and RE of HIV-infected individuals with and w/o cART.

It was shown that the TI contains more cell-associated HIV DNA than the RE, and is, therefore, the most important anatomical reservoir during chronic HIV infection. These particular findings are consistent with previous work of ours [22], which investigated the role of PD-1 in fueling cellular HIV-1 reservoirs [22]. However, the role of CXCR3 and IP-10 in this issue is the subject of the present study.

In our study, the lowest number of T_CD4_ occurred (i) in HIV+ compared to CTRL, (ii) in the GALT compared to the PB (Figure 1a), and (iii) in HIV^+^_NAIVE_ compared to HIV^+^_cART_. These findings confirm that effective combined antiretroviral therapy (cART) restores the T_CD4_ count primarily in the PB. In contrast, no complete recovery of T_CD4_ was observed in the TI, despite cART (Figure 1a). In addition, we showed that the GALT—reflecting increased immune activation—harbored significantly (i) more memory CD4+ T cells (T_M_) and (ii) fewer antigen-naive T cells (T_N_) compared to the PB (Figure 1b). These observations are consistent with previous work by us and others [30,31,32,33], and highlights the crucial role of the TI in HIV pathogenesis and HIV persistence in the GALT, despite cART. In contrast to previous studies, in which T_CM_ was the most frequent subset in the PB and T_EM_ in TI [30,34], T_TM_ was the most frequent subset in our study **(**Figure 1c). Distinct (i) clinical characteristics, (ii) gating strategy or (iii) techniques may explain the contrast observed. Despite cART for a median duration of eight years (IQR 5–11), more HIV DNA was detected (i) in the GALT, when compared to the PB, and (ii) in the TI, when compared to the PB and RE (TI > RE = PB). Previous studies had already shown that the GALT harbored more HIV DNA when compared to the PB [35,36,37,38].

Distinct cellular subsets (T_CD4_: T_M_, T_CM_, T_EM_ and T_TM_) were examined, as antigenic proliferation and maturation alter the qualitative features of potential HIV reservoirs. Regarding CXCR3 expression on these subsets, and IP-10 levels in the blood and gut, the role of the TI as the most decisive HIV reservoir is emphasized. CXCR3 patterns and IP-10 levels had already been associated with the establishment of HIV reservoirs in the intestine of non-human primates [14], as well as in the peripheral blood [19], lymph nodes [39] and duodenum [40] of HIV-positive patients. Here, these findings were described for the first time in the cellular HIV reservoir in the terminal ileum and rectum of the human GALT. In detail, CXCR3 expressions were higher (i) in HIV+ when compared to the healthy controls, (ii) in the GALT when compared to the PB, (iii) on T_M_ when compared to T_N_, and (iv) on T_CM_ when compared to T_EM_ or T_TM_, in all three sites (Figure 2a,b).

Next, we tested whether CXCR3 expression correlated with cell-associated HIV DNA in the TI, as CXCR3 marks blood memory CD4+ T cells that contain replication-competent virus [41], and is associated with chronic immune activation in human blood [41]. In particular, T_CM_ is considered a significant replication-competent HIV-1 reservoir [34], and, in our study, it showed the highest CXCR3 expression in all sites (Figure 2b). However, we observed no links between CXCR3 expression, on either blood or ileal memory T cells, and HIV DNA (Figure 2d,f). These findings are in line with previous studies, in which CCR6+Th17 subsets predominantly contribute to HIV persistence in the PB [39,42,43], lymph nodes [39] and colon [43] during cART.

Similarly, in the PB, the levels of CXCR3’s proinflammatory ligand IP-10 were increased in treatment-naïve HIV+ compared to healthy and cART-treated individuals (Figure 2c). By contrast, in the TI, the IP-10 levels were similar in treatment-naïve and cART-treated HIV+. As in our observations with HIV DNA, cART reduces IP-10 more effectively in the PB than in the TI. Given that elevated IP-10 levels were found to be associated with CD4+ T cell decline and an increased risk of rapid progression towards AIDS [14], the reduction in blood IP-10 levels may signal successful antiretroviral treatment. During primary HIV infection, systemic IP-10 levels correlate with cell-associated HIV DNA in the PB and semen [14,20]. Exposure to IP-10 allows efficient HIV-1 nuclear localization and integration of the HIV-1 provirus [18], characterizing the HIV reservoir. We, thus, tested for correlations of IP-10 levels and CXCR3 expression with HIV DNA (Figure 2d–g). In our study, neither CXCR3 nor IP-10 levels were indicative of the size of the cellular HIV reservoir in the TI or RE (Figure 2d–g). This could have several reasons. For instance, the majority of our patients were in the chronic rather than acute phase of infection. Another reason might be that in chronic, treated infection, other parameters, such as persisting tissue damage or the metabolic state of the cell, have a stronger impact on the HIV reservoir size than local IP-10 levels [13,44,45]. While we measured serum levels in the PB, gut IP-10 levels arose from the ongoing production of collected biopsies. Although this method does not allow for a comparison between sites, it does allow for a valid comparison between patient groups within a single site. However, the total HIV DNA was measured in cell lysates, so we cannot provide any information about the type of DNA and its replication ability. Measuring the total cell-associated HIV DNA is only one of many imperfect surrogates of HIV-1 reservoirs. Other limitations of our study were (i) the small sample size, (ii) few biopsies and, thus, limited cell count, (iii) low cell yield, and (iv) no viral outgrowth assay. Nevertheless, the increase in CXCR3 expression observed in both the TI and PB indicates CXCR3-mediated processes that are stimulating or impairing, and require further investigation. Whether CXCR3 is a competent indicator for replication-competent HIV reservoirs will have to be examined in future qualitative analyses of the HIV reservoir of the GALT.

In conclusion, our study highlights (i) the role of the TI as an important anatomic sanctuary for HIV, (ii) the failure of cART to eliminate HIV DNA, IP-10, and CXCR3 expression in the TI, (iii) that neither CXCR3 expression nor IP-10 levels are indicative of the size of the cellular reservoir determined by the amount of HIV DNA, and (iv) CXCR3-mediated processes that affect HIV persistence in the GALT. The crucial role of the TI as an anatomical HIV reservoir had been significantly underrepresented in studies to date. Despite the difficult accessibility of the TI, the analysis of samples from the TI is crucial for understanding HIV immunopathogenesis, to develop translational intervention strategies for HIV-1 eradication and for the control of viral HIV reservoirs in the future. In our study, neither CXCR3 expression nor IP-10 levels were indicative of the quantitative size of the cellular HIV reservoir in the blood and GALT (TI and RE), as determined by the amount of HIV DNA. Future research will have to investigate whether CXCR3 and IP-10 qualitatively allow us to find replication-competent HIV reservoirs in the human GALT.

## 4. Materials and Methods

### 4.1. Resources

After informed consent, 23 mL of PB, as well as six to ten ileal and rectal biopsies from colonoscopy, were obtained from 18 cART-treated (HIV^+^_cART_) and 6 treatment-naïve (HIV^+^_NAIVE_) HIV+ patients, and 16 healthy individuals, from the (i) University Hospital Cologne, (ii) Augustinerinnen Hospital, Cologne, and (iii) PanKlinik—Clinic for Coloproctology, Cologne. All biomaterials were collected at the University Hospital Cologne.

Inclusion criteria: 18–65 years old and infection with HIV-1. Exclusion criteria: contraindications for sedation or endoscopy and inflammatory bowel diseases. Clinical characteristics were documented (Table 1). The ileal and rectal biopsies from colonoscopy were immediately placed in 5 mL tissue culture medium (RPMI 1640, F 1215, Biochrom, Cambridge, UK), equipped with 10% penicillin/streptomycin and 25 µg/mL amphotericin B, used as supernatant after removal of biopsies.

### 4.2. Cell Isolation and FACS Sorting

Density centrifugation on a Ficoll gradient (L 6115, Biochrom, Cambridge, UK) was used to separate peripheral blood mononuclear cells (PBMCs) and lamina propria mononuclear cells (LPMCs), as described by Lehmann et al. [46], and were kept frozen at −180 °C. Thawed mononuclear cells were stained (CD3-APC-H7 (1:40, 560176, Becton Dickinson (BD), Franklin Lakes, NJ, USA), CD4-FITC (1:40, 555346, BD, Franklin Lakes, NJ, USA), CD45RO APC (1:10, 130-109-430, Miltenyi Biotec, Bergisch Gladbach, Germany), CCR7 Pe-Cy7 (1:80, 557648, BD, Franklin Lakes, NJ, USA), CD27 PE (1:40, 560985, BD, Franklin Lakes, NJ, USA) and CXCR3-BV510 (1:20, 353726, Biolegend, San Diego, CA, USA) and expression of CXCR3 (gMFI, geometric mean fluorescence intensities) on sorted CD4+CD45RO+ memory T-cell subsets (T_M_) was assessed (T_CM_, T central memory: CD27+CCR7+; T_TM_, T transitional memory: CD27+CCR7−; and T_EM_, T effector memory cells: CD27−CCR7−) (Figure 1b). Exemplary FACS plots are accessible in previous work of ours [22]. Samples were sorted on a BD (Franklin Lakes, NJ, USA) FACSAria Fusion flow cytometer into 2 mL collection tubes at 4-way purity and incubated without prior culturing with the following antibodies. Isolation did not affect the detection of membrane proteins by antibodies. Flow cytometry was performed on a BD (Franklin Lakes, NJ, USA) FACSAria Fusion flow cytometer.

### 4.3. DNA Extraction and HIV-1 Quantification

Total HIV DNA was extracted from sorted memory CD4+ T cell subsets using QIAamp DNA Blood Kit (51104, Qiagen, Hilden, Germany), according to the manufacturer’s instructions. HIV copies of the eluate were quantified using the Versant HIV-1 RNA 1.5 Assay (kPCR) from Siemens (Munich, Germany), with a range of quantification of 37 to 11 × 10^6^ copies/mL. Cell-associated HIV-1 DNA copies/mL were normalized to β-globin levels and expressed as HIV copies per T_M_ × 10^6^.

### 4.4. Ultrasensitive IP-10 Quantification

Ultrasensitive digital ELISA (Simoa; Quanterix, Billerica, MA, USA; pg/mL) was performed on thawed serum and gut tissue supernatants to quantify IP-10 levels as previously described [47]. Gut biopsies remained for, on average, 4 h in tissue culture medium, which was later used for ultrasensitive IP-10 quantification.

### 4.5. Statistical Analyses

Statistical analysis was conducted with GraphPad Prism Software Version 9.3.1. (GraphPad Software, La Jolla, CA, USA). Significant differences (*p*-values < 0.05) were tested with a two-tailed Mann-Whitney test or Wilcoxon matched-pairs test, as applicable. Spearman’s r was used to describe non-parametric correlations. Correlation analyses were performed in HIV^+^_cART_ only. All values are represented as the median with interquartile ranges (IQR), unless otherwise stated.

## Figures and Tables

**Table 1:** Clinical characteristics of cART-treated and treatment-naive HIV-positive individuals (HIV+) and healthy controls (CTRL).

Patients	n	Age, (Years)	HIV-1, (Years)	cART, (Years)	CD4^+^, (Cells/mm^3^)	CD4^+^, (%)	CD4^+^ Nadir, (Cells/mm^3^)	Plasma Viral Load, (RNA Copies/mL)	Aviremia, (Years)
HIV^+^_cART_	18_(16♂, 2♀︎)_	55_(IQR 47–61)_	14_(IQR 11–20)_	8_(IQR 5–10)_	745_(IQR 464–973)_	33_(IQR 27–36)_	280_(IQR 197–423)_	20_(IQR 20–40)_	8_(IQR 6–14)_
HIV^+^_NAIVE_	6_(6♂, 0♀︎)_	49_(IQR 42–50)_	4_(IQR 2–14)_	0	70_(IQR 20–270)_	16_(IQR 3–30)_	40_(IQR 20–270)_	792k_(IQR 17 k–3.7 M)_	0
CTRL	16_(7♂, 9♀)_	58_(IQR 49–65)_	n.a.	n.a.	1075_(IQR 952–1289)_	35_(IQR 33–36)_	n.a.	n.a.	n.a.

cART, combined antiretroviral therapy; HIV, human immunodeficiency virus; HIV^+^, HIV-positive individuals; HIV^+^_cART_, cART-treaded HIV^+^; HIV^+^_NAIVE_, treatment-naive HIV^+^; n, number; ♂, men; ♀︎, women; CD4^+^, CD4-positive T cells; RNA, ribonucleic acid; mL, milliliters; IQR, interquartile range; k, thousand; M, million; n.a., not applicable.

## Data Availability

Raw data were generated at the Medical Faculty and University Hospital Cologne. Derived data supporting the findings of this study are available from the corresponding authors on request.

## References

[1] Finzi D., Blankson J., Siliciano J.D., Margolick J.B., Chadwick K., Pierson T., Smith K., Lisziewicz J., Lori F., Flexner C. (1999). Latent infection of CD4+ T cells provides a mechanism for lifelong persistence of HIV-1, even in patients on effective combination therapy. Nat. Med..

[2] Mowat A.M., Agace W.W. (2014). Regional specialization within the intestinal immune system. Nat. Rev. Immunol..

[3] Cornes J.S. (1965). Number, size, and distribution of Peyer’s patches in the human small intestine: Part I The development of Peyer’s patches. Gut.

[4] Rueda C.M., Velilla P.A., Chougnet C.A., Montoya C.J., Rugeles M.T. (2012). HIV-induced T-cell activation/exhaustion in rectal mucosa is controlled only partially by antiretroviral treatment. PLoS ONE.

[5] Khoury G., Fromentin R., Solomon A., Hartogensis W., Killian M., Hoh R., Somsouk M., Hunt P.W., Girling V., Sinclair E. (2017). Human Immunodeficiency Virus Persistence and T-Cell Activation in Blood, Rectal, and Lymph Node Tissue in Human Immunodeficiency Virus-Infected Individuals Receiving Suppressive Antiretroviral Therapy. J. Infect. Dis..

[6] Hatano H., Somsouk M., Sinclair E., Harvill K., Gilman L., Cohen M., Hoh R., Hunt P.W., Martin J.N., Wong J.K. (2013). Comparison of HIV DNA and RNA in gut-associated lymphoid tissue of HIV-infected controllers and noncontrollers. AIDS.

[7] Asmuth D.M., Ma Z.M., Mann S., Knight T.H., Yotter T., Albanese A., Melcher G.P., Troia-Cancio P., Hayes T., Miller C.J. (2012). Gastrointestinal-associated lymphoid tissue immune reconstitution in a randomized clinical trial of raltegravir versus non-nucleoside reverse transcriptase inhibitor-based regimens. AIDS.

[8] Descours B., Petitjean G., López-Zaragoza J.-L., Bruel T., Raffel R., Psomas C., Reynes J., Lacabaratz C., Levy Y., Schwartz O. (2017). CD32a is a marker of a CD4 T-cell HIV reservoir harbouring replication-competent proviruses. Nature.

[9] Pérez L., Anderson J., Chipman J., Thorkelson A., Chun T.-W., Moir S., Haase A.T., Douek D.C., Schacker T.W., Boritz E.A. (2018). Conflicting evidence for HIV enrichment in CD32+ CD4 T cells. Nature.

[10] Bertagnolli L.N., White J.A., Simonetti F.R., Beg S.A., Lai J., Tomescu C., Murray A.J., Antar A.A.R., Zhang H., Margolick J.B. (2018). The role of CD32 during HIV-1 infection. Nature.

[11] Abdel-Mohsen M., Kuri-Cervantes L., Grau-Exposito J., Spivak A.M., Nell R.A., Tomescu C., Vadrevu S.K., Giron L.B., Serra-Peinado C., Genesca M. (2018). CD32 is expressed on cells with transcriptionally active HIV but does not enrich for HIV DNA in resting T cells. Sci. Transl. Med..

[12] Darcis G., Kootstra N.A., Hooibrink B., van Montfort T., Maurer I., Groen K., Jurriaans S., Bakker M., van Lint C., Berkhout B. (2020). CD32(+)CD4(+) T Cells Are Highly Enriched for HIV DNA and Can Support Transcriptional Latency. Cell Rep..

[13] Valle-Casuso J.C., Angin M., Volant S., Passaes C., Monceaux V., Mikhailova A., Bourdic K., Avettand-Fenoel V., Boufassa F., Sitbon M. (2019). Cellular Metabolism Is a Major Determinant of HIV-1 Reservoir Seeding in CD4+ T Cells and Offers an Opportunity to Tackle Infection. Cell Metab..

[14] Ploquin M.J., Madec Y., Casrouge A., Huot N., Passaes C., Lecuroux C., Essat A., Boufassa F., Jacquelin B., Jochems S.P. (2016). Elevated Basal Pre-infection CXCL10 in Plasma and in the Small Intestine after Infection Are Associated with More Rapid HIV/SIV Disease Onset. PLoS Pathog..

[15] Khoury G., Anderson J.L., Fromentin R., Hartogenesis W., Smith M.Z., Bacchetti P., Hecht F.M., Chomont N., Cameron P.U., Deeks S.G. (2016). Persistence of integrated HIV DNA in CXCR3 + CCR6 + memory CD4+ T cells in HIV-infected individuals on antiretroviral therapy. AIDS.

[16] Gosselin A., Wiche Salinas T.R., Planas D., Wacleche V.S., Zhang Y., Fromentin R., Chomont N., Cohen E.A., Shacklett B., Mehraj V. (2017). HIV persists in CCR6+CD4+ T cells from colon and blood during antiretroviral therapy. AIDS.

[17] Wang C., Kang S.G., Lee J., Sun Z., Kim C.H. (2009). The roles of CCR6 in migration of Th17 cells and regulation of effector T-cell balance in the gut. Mucosal. Immunol..

[18] Cameron P.U., Saleh S., Sallmann G., Solomon A., Wightman F., Evans V.A., Boucher G., Haddad E.K., Sekaly R.P., Harman A.N. (2010). Establishment of HIV-1 latency in resting CD4+ T cells depends on chemokine-induced changes in the actin cytoskeleton. Proc. Natl. Acad. Sci. USA.

[19] Banga R., Procopio F.A., Ruggiero A., Noto A., Ohmiti K., Cavassini M., Corpataux J.-M., Paxton W.A., Pollakis G., Perreau M. (2018). Blood CXCR3+ CD4 T Cells Are Enriched in Inducible Replication Competent HIV in Aviremic Antiretroviral Therapy-Treated Individuals. Front. Immunol..

[20] Cheret A., Durier C., Melard A., Ploquin M., Heitzmann J., Lecuroux C., Avettand-Fenoel V., David L., Pialoux G., Chennebault J.M. (2017). Impact of early cART on HIV blood and semen compartments at the time of primary infection. PLoS ONE.

[21] Lane B.R., King S.R., Bock P.J., Strieter R.M., Coffey M.J., Markovitz D.M. (2003). The C-X-C chemokine IP-10 stimulates HIV-1 replication. Virology.

[22] Horn C., Augustin M., Ercanoglu M.S., Heger E., Knops E., Bondet V., Duffy D., Chon S.H., Nierhoff D., Oette M. (2021). HIV DNA reservoir and elevated PD-1 expression of CD4 T-cell subsets particularly persist in the terminal ileum of HIV-positive patients despite cART. HIV Med..

[23] Vidya Vijayan K.K., Karthigeyan K.P., Tripathi S.P., Hanna L.E. (2017). Pathophysiology of CD4+ T-Cell Depletion in HIV-1 and HIV-2 Infections. Front Immunol.

[24] Guadalupe M., Reay E., Sankaran S., Prindiville T., Flamm J., McNeil A., Dandekar S. (2003). Severe CD4+ T cell depletion in gut lymphoid tissue during primary human immunodeficiency virus type 1 infection and substantial delay in restoration following highly active antiretroviral therapy. J. Virol..

[25] Mehandru S., Poles M.A., Tenner-Racz K., Horowitz A., Hurley A., Hogan C., Boden D., Racz P., Markowitz M. (2004). Primary HIV-1 infection is associated with preferential depletion of CD4+ T lymphocytes from effector sites in the gastrointestinal tract. J. Exp. Med..

[26] Brenchley J.M., Schacker T.W., Ruff L.E., Price D.A., Taylor J.H., Beilman G.J., Nguyen P.L., Khoruts A., Larson M., Haase A.T. (2004). CD4+ T cell depletion during all stages of HIV disease occurs predominantly in the gastrointestinal tract. J. Exp. Med..

[27] Douek D.C., Roederer M., Koup R.A. (2009). Emerging concepts in the immunopathogenesis of AIDS. Annu. Rev. Med..

[28] Brenchley J.M., Price D.A., Douek D.C. (2006). HIV disease: Fallout from a mucosal catastrophe?. Nat. Immunol..

[29] Mehandru S., Dandekar S. (2008). Role of the gastrointestinal tract in establishing infection in primates and humans. Curr. Opin. HIV AIDS.

[30] Yukl S.A., Shergill A.K., Ho T., Killian M., Girling V., Epling L., Li P., Wong L.K., Crouch P., Deeks S.G. (2013). The distribution of HIV DNA and RNA in cell subsets differs in gut and blood of HIV-positive patients on ART: Implications for viral persistence. J. Infect. Dis..

[31] Hayes T.L., Asmuth D.M., Critchfield J.W., Knight T.H., McLaughlin B.E., Yotter T., McConnell D.H., Garcia J.C., Pollard R.B., Shacklett B.L. (2013). Impact of highly active antiretroviral therapy initiation on CD4(+) T-cell repopulation in duodenal and rectal mucosa. AIDS.

[32] Mehandru S., Poles M.A., Tenner-Racz K., Jean-Pierre P., Manuelli V., Lopez P., Shet A., Low A., Mohri H., Boden D. (2006). Lack of mucosal immune reconstitution during prolonged treatment of acute and early HIV-1 infection. PLoS Med..

[33] Macal M., Sankaran S., Chun T.W., Reay E., Flamm J., Prindiville T.J., Dandekar S. (2008). Effective CD4+ T cell restoration in gut-associated lymphoid tissue of HIV-infected patients is associated with enhanced Th17 cells and polyfunctional HIV-specific T-cell responses. Mucosal. Immunol..

[34] Chomont N., El-Far M., Ancuta P., Trautmann L., Procopio F.A., Yassine-Diab B., Boucher G., Boulassel M.R., Ghattas G., Brenchley J.M. (2009). HIV reservoir size and persistence are driven by T cell survival and homeostatic proliferation. Nat. Med..

[35] Josefsson L., von Stockenstrom S., Faria N.R., Sinclair E., Bacchetti P., Killian M., Epling L., Tan A., Ho T., Lemey P. (2013). The HIV-1 reservoir in eight patients on long-term suppressive antiretroviral therapy is stable with few genetic changes over time. Proc. Natl. Acad. Sci. USA.

[36] Chun T.W., Nickle D.C., Justement J.S., Meyers J.H., Roby G., Hallahan C.W., Kottilil S., Moir S., Mican J.M., Mullins J.I. (2008). Persistence of HIV in gut-associated lymphoid tissue despite long-term antiretroviral therapy. J. Infect. Dis..

[37] Moron-Lopez S., Puertas M.C., Galvez C., Navarro J., Carrasco A., Esteve M., Manye J., Crespo M., Salgado M., Martinez-Picado J. (2017). Sensitive quantification of the HIV-1 reservoir in gut-associated lymphoid tissue. PLoS ONE.

[38] Yukl S.A., Gianella S., Sinclair E., Epling L., Li Q., Duan L., Choi A.L., Girling V., Ho T., Li P. (2010). Differences in HIV burden and immune activation within the gut of HIV-positive patients receiving suppressive antiretroviral therapy. J. Infect. Dis..

[39] Wacleche V.S., Goulet J.P., Gosselin A., Monteiro P., Soudeyns H., Fromentin R., Jenabian M.A., Vartanian S., Deeks S.G., Chomont N. (2016). New insights into the heterogeneity of Th17 subsets contributing to HIV-1 persistence during antiretroviral therapy. Retrovirology.

[40] Loiseau C., Requena M., Nayrac M., Mavigner M., Cazabat M., Iscache A.L., Carrere N., Suc B., Alric L., Izopet J. (2019). Increased CXCR3+ T Cells Impairs Recruitment of T-Helper Type 17 Cells via Interferon gamma and Interleukin 18 in the Small Intestine Mucosa During Treated HIV-1 Infection. J. Infect. Dis..

[41] Cecchinato V., Bernasconi E., Speck R.F., Proietti M., Sauermann U., D’Agostino G., Danelon G., Rezzonico Jost T., Grassi F., Raeli L. (2017). Impairment of CCR6+ and CXCR3+ Th Cell Migration in HIV-1 Infection Is Rescued by Modulating Actin Polymerization. J. Immunol..

[42] Cleret-Buhot A., Zhang Y., Planas D., Goulet J.P., Monteiro P., Gosselin A., Wacleche V.S., Tremblay C.L., Jenabian M.A., Routy J.P. (2015). Identification of novel HIV-1 dependency factors in primary CCR4(+)CCR6(+)Th17 cells via a genome-wide transcriptional approach. Retrovirology.

[43] Seddiki N., Zaunders J., Phetsouphanh C., Brezar V., Xu Y., McGuire H.M., Bailey M., McBride K., Hey-Cunningham W., Munier C.M.L. (2021). CD73(+) CD127(high) Long-Term Memory CD4 T Cells Are Highly Proliferative in Response to Recall Antigens and Are Early Targets in HIV-1 Infection. Int. J. Mol. Sci..

[44] Gandhi R.T., McMahon D.K., Bosch R.J., Lalama C.M., Cyktor J.C., Macatangay B.J., Rinaldo C.R., Riddler S.A., Hogg E., Godfrey C. (2017). Levels of HIV-1 persistence on antiretroviral therapy are not associated with markers of inflammation or activation. PLoS Pathog..

[45] Fromentin R., DaFonseca S., Costiniuk C.T., El-Far M., Procopio F.A., Hecht F.M., Hoh R., Deeks S.G., Hazuda D.J., Lewin S.R. (2019). PD-1 blockade potentiates HIV latency reversal ex vivo in CD4(+) T cells from ART-suppressed individuals. Nat. Commun..

[46] Lehmann C., Jung N., Forster K., Koch N., Leifeld L., Fischer J., Mauss S., Drebber U., Steffen H.M., Romerio F. (2014). Longitudinal analysis of distribution and function of plasmacytoid dendritic cells in peripheral blood and gut mucosa of HIV infected patients. J. Infect. Dis..

[47] Decalf J., Tarbell K.V., Casrouge A., Price J.D., Linder G., Mottez E., Sultanik P., Mallet V., Pol S., Duffy D. (2016). Inhibition of DPP4 activity in humans establishes its in vivo role in CXCL10 post-translational modification: Prospective placebo-controlled clinical studies. EMBO Mol. Med..

